# An end-to-end framework for private DGA detection as a service

**DOI:** 10.1371/journal.pone.0304476

**Published:** 2024-08-28

**Authors:** Ricardo J. M. Maia, Dustin Ray, Sikha Pentyala, Rafael Dowsley, Martine De Cock, Anderson C. A. Nascimento, Ricardo Jacobi

**Affiliations:** 1 Department of Computer Science, University of Brasilia, Federal District, Brasília, Brazil; 2 School of Engineering, University of Washington Tacoma, Tacoma, Washington, United States of America; 3 Department of Software Systems and Cybersecurity, Monash University, Melbourne, Australia; 4 Department of Applied Mathematics, Computer Science and Statistics, Ghent University, Ghent, Belgium; 5 Visa Research (Work done while at University of Washington Tacoma), Foster City, California, United States of America; Government College University Lahore, PAKISTAN

## Abstract

Domain Generation Algorithms (DGAs) are used by malware to generate pseudorandom domain names to establish communication between infected bots and command and control servers. While DGAs can be detected by machine learning (ML) models with great accuracy, offering DGA detection as a service raises privacy concerns when requiring network administrators to disclose their DNS traffic to the service provider. The main scientific contribution of this paper is to propose the first end-to-end framework for privacy-preserving classification as a service of domain names into DGA (malicious) or non-DGA (benign) domains. Our framework achieves these goals by carefully designed protocols that combine two privacy-enhancing technologies (PETs), namely secure multi-party computation (MPC) and differential privacy (DP). Through MPC, our framework enables an enterprise network administrator to outsource the problem of classifying a DNS (Domain Name System) domain as DGA or non-DGA to an external organization without revealing any information about the domain name. Moreover, the service provider’s ML model used for DGA detection is never revealed to the network administrator. Furthermore, by using DP, we also ensure that the classification result cannot be used to learn information about individual entries of the training data. Finally, we leverage post-training float16 quantization of deep learning models in MPC to achieve efficient, secure DGA detection. We demonstrate that by using quantization achieves a significant speed-up, resulting in a 23% to 42% reduction in inference runtime without reducing accuracy using a three party secure computation protocol tolerating one corruption. Previous solutions are not end-to-end private, do not provide differential privacy guarantees for the model’s outputs, and assume that model embeddings are publicly known. Our best protocol in terms of accuracy runs in about 0.22s.

## 1 Introduction

Malicious software (malware) is the class of software that infects computers to perform unauthorized actions in the system or gain unauthorized access to information. Malware is a highly significant source of illicit activities with increasing impact [1–4], and producing substantial losses in sectors such as the government, energy, and manufacturing [5]. Examples of malware families include trojan horses, viruses, ransomware, key loggers, worms, spyware, and hidden cryptominers. Some common objectives of these types of malware are information or identity theft, espionage, and service disruption [1, 4].

Botnets, i.e., computer networks infected by malware, are commonly controlled, operated, and updated through communicating with a Command and Control (C&C) server that is under the control of an adversary or botmaster [6]. When the IP address of the C&C server is hard-coded directly into the malware, intrusion detection systems (IDS) or firewalls on Domain Name System (DNS) servers can blacklist the detected malicious domain names and block the connection to the C&C server, effectively rendering the malware useless. Cyber-attackers have, therefore, adopted innovative techniques for obfuscating and concealing the C&C’s identity. Among the most prevalent approaches are Domain Generation Algorithms (DGA) [7].

DGAs are algorithms that periodically generate pseudorandom combinations of characters or words to form hundreds or even thousands of new domain names. The key idea is that DGAs can generate the same set of new domain names when executed by two different machines, such as a botmaster and an infected machine. The botmaster registers one or more generated domain names, while the infected machines systematically query the domains from the generated list until one of them is resolved. The domains from the list that the botmaster has not registered will typically result in a non-existent domain response when queried and can be discarded by the infected machine. Once an infected machine queries a registered domain name, communication between the infected bot and the C&C center is established, and malicious activities, as instructed by the C&C center, can be performed by the bot.

The constant changes to the domain name of the C&C server make it much more difficult for IDS and firewalls to detect and contain the attacks. The challenge of mitigating attacks that use DGA techniques lies in identifying malicious domain names. The DNS server must be able to detect and block malicious domain names while keeping normal operations for benign domain names. In short, the ability to identify malicious domains can drastically decrease the harm caused by malware.

Using machine learning models to create classifiers that can identify and separate benign domains from DGA-generated malware domains is a viable approach (see [8] and references therein). Such classifiers (models) can be deployed as automatic malware detection systems in enterprise networks. The state-of-the-art models use deep learning techniques that achieve high accuracy but require large amounts of training data [9]. Due to this data demand, models are usually available with third-party organizations (service providers) as a DGA detection service where the DNS traffic of an enterprise is sent to the service provider who then classifies the incoming traffic into malicious or benign domains and sends the classification result to the enterprise [10]. Such an outsourced DGA detection as a service model presents numerous privacy challenges. The DNS traffic of an enterprise holds sensitive information that can impact the privacy of all the enterprise network users, which raises privacy concerns for the users in the above DGA detection as a service paradigm. A potential solution to address this concern is to make the ML model used for DGA detection available to enterprise network administrators to deploy it locally. This is, again, problematic as the model is proprietary to the service provider. Moreover, the data used for training such models can be private, and releasing the model to the enterprises renders the underlying training data vulnerable to attacks [11, 12]. Simultaneously protecting the sensitive data of the enterprise and the service provider is a significant challenge.

In this paper, we investigate the possibility of simultaneously providing privacy for the machine learning model holder and the party interested in performing the domain classification. Our paper proposes an end-to-end privacy-preserving framework for outsourced DGA classification that preserves enterprise users’ and service providers’ privacy. Our result allows network administrators to outsource traffic analysis (in this case, DNS traffic analysis) to outside parties so that no information about the DNS traffic is ever leaked. It also protects the intellectual property of the model holder, ensuring the computing parties cannot obtain any information on the model.

### Scientific contributions

We propose a novel framework that provides the benefits of automated and outsourced DGA detection while preserving the privacy of enterprise network users’ and DGA detection service providers’ data. In our framework, neither the DNS traffic is released in the clear to the DGA detection service providers nor is the model made available to the enterprise network administrators (no private data is revealed to any other party). Furthermore, we incorporate techniques that prevent attackers from gaining additional information about the training data or reconstructing the model from the classification results sent to the enterprise network administrators.

**Fig 1:**
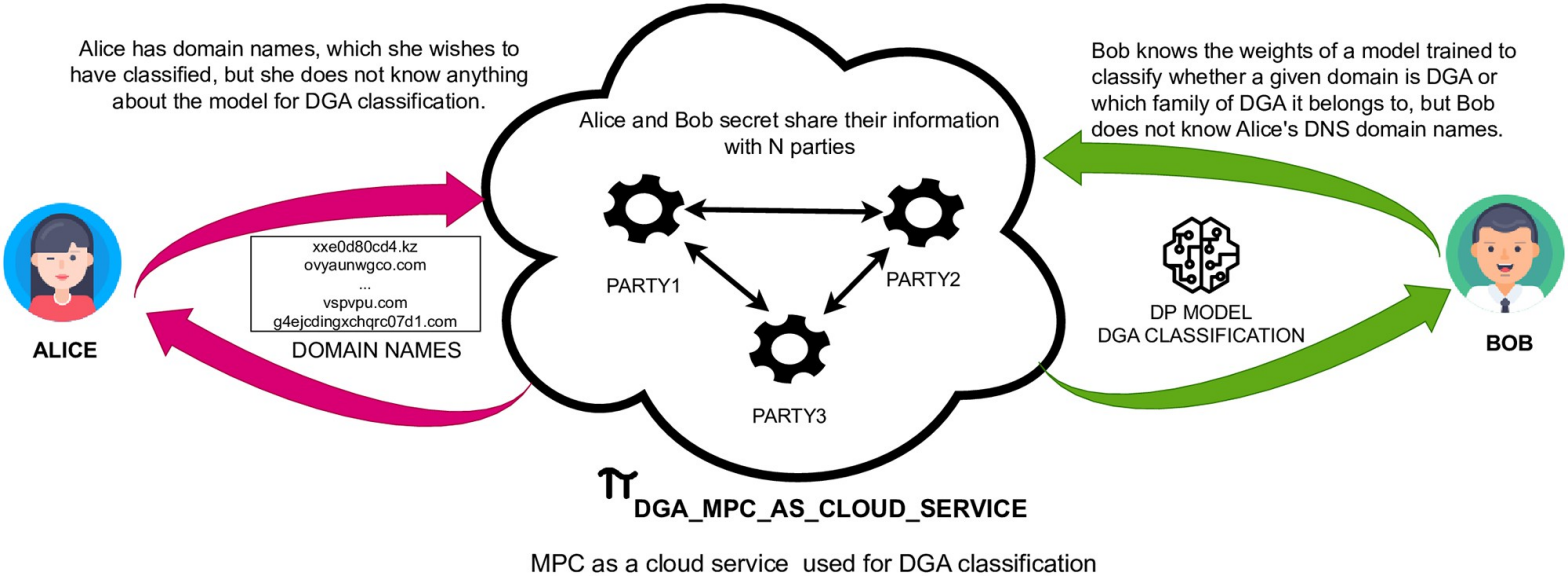
Flow diagram illustrating end-to-end privacy-preserving DGA detection as a service. The DNS domain name classifier of the service provider (Bob) is trained with DP-SGD to provide differential privacy (DP) guarantees (output privacy). New domain names coming from Alice are classified with Bob’s model by secure multi-party computation (MPC). Protocols executed by MPC servers in the cloud over encrypted data (input privacy).

Fig 1 illustrates our framework at a high level. *Alice* represents the enterprise and holds the DNS domains and traffic. *Bob* represents the service provider and has the weights (parameters) of the trained machine learning model.*Bob* can choose to train a multilayer perceptron (MLP), a one-dimensional convolutional neural network (1D-CNN), or a long short-term memory (LSTM), and the chosen model shall be trained with differential privacy guarantees [13]. Furthermore, *Alice* wants her DNS traffic classified by *Bob*. We hereafter refer to the *Alice’s* DNS data and *Bob’s* model weights as private data.

Our proposed framework employs techniques from secure multi-party computation [14] to preserve input privacy. To this end, *Alice* and *Bob* secret share their private data with a set of untrusted computational servers (parties). The MPC servers perform computations on the secret shares to label domain names as benign or malicious in a way such that:

(P1) No individual MPC server should obtain any information about *Alice’s* domain names;
(P2) No individual MPC server should learn any information about the weights of *Bob’s* machine learning model;
(P3) The result of the classification should be revealed only to *Alice*;
(P4) The result of the classification should reveal no private information about individual entries in *Bob’s* training data set to *Alice*.

We note that (P4) provides *output privacy* and is achieved as *Bob* has trained the model with DP guarantees. (P1)–(P3) provide *input privacy* and are achieved through the novel MPC protocols that we propose for inference with a neural network model trained for DGA detection. MPC protocols usually lead to high communication and computation costs, thus impacting the inference runtime and overall performance. To improve the performance of our proposed MPC protocols for secure classification of DGA domains, we leverage the benefits of quantization schemes available in TensorFlow (TFLite). The quantization method reduces the precision of a machine learning model’s parameters, typically 32-bit floating point numbers [15]. We propose that *Bob* uses post-training quantization techniques on the DP-trained model before using the model for classification.

The closest related work [16] to ours on privacy-preserving detection of DGAs provides only input privacy through MPC, leaving the inference phase vulnerable to privacy attacks on the training data (see Section 2 for more details). We propose the first end-to-end privacy-preserving framework for DGA classification, ensuring input and output privacy. We summarize our contributions below:

- We propose a novel framework for private classification as a service of domains into DGA/non-DGA, with input privacy (MPC) and output privacy (DP) guarantees.
- Our proposed framework is the first that considers differentially private training of models for DGA classification.
- Our proposed solution works with classifiers based on multilayer perceptrons, 1D convolutional neural networks, and long short-term memory networks. Our MPC protocol for LSTM is novel, efficient, and the first ever implemented in the MP-SPDZ MPC framework [17].
- We evaluate our framework on real datasets—DGArchive and Alexa—for binary and multiclass domain name classification tasks. The binary classification problem distinguishes a domain as benign and malicious. The multiclass classification problem also outputs the DGA family corresponding to the malicious domain.
- We empirically analyze our approach’s privacy and utility trade-offs when using MLP, 1D-CNN, and LSTM. We observe that providing output privacy degrades the accuracy by a small amount in our experiments due to the noise introduced to provide DP guarantees. Using our MPC protocols to provide input privacy, on the other hand, does not degrade the utility of the classification model. The fastest model in terms of the runtime is a multilayer perceptron with an inference time equal to 0.07s. The model with the best accuracy is 1D-CNN, achieving an accuracy equal to 93% for an epsilon 5.
- We demonstrate the efficiency of our proposed solution in terms of runtime with 2 or 3 computing parties.
  With quantization, we observe significant improvements in the performance of our MPC protocols, leading to reduced communication rounds and inference time. Our experiments show a 23% to 42% improvement in inference runtime without affecting accuracy in the 3PC setting (using replicated secret sharing). This demonstrates that we can achieve near real-time secure detection of DGA domains.

We present now how this paper is organized. Section 2 covers related works. Section 3 covers preliminaries related to machine learning, cryptography, and differential privacy. Section 4 describes the proposed framework, security requirements, and threat model. The proposed MPC protocols for embedding, MLP, 1D-CNN, and LSTM and the discussion on post-training float16 quantization are in section 5. Details about the results and experiment are in section 6, which discusses the experiments’ Security and Privacy, Utility-Privacy Trade-Off, and runtime metrics.

## 2 Related works

We now present the related literature and compare our solution to previous ones.

### 2.1 DGA detection with deep learning

DGA detection methods did not rely on machine learning in the early stages. For instance, Sharifnya et al. [18] developed a technique that identifies hosts with a high volume of failed DNS queries, subsequently adding these hosts to a “suspicious failure matrix.” This section lists relevant works regarding DGA detection using deep or machine learning without any privacy guarantees.

Li et al. [19] propose several real-time detection models and frameworks that utilize meta-data generated from domains and combine the advantages of a deep neural network model and a lexical features-based model using the ensemble technique. Another work in this line is [20], which uses Helix’s architecture that represents DGA as embeddings. The utilization of multilayer perceptron for DGA output detection was also investigated [21, 22].

Huang et al. [23] propose a Helios architecture that uses CNN to detect DGA. Zhou et al. [24] also uses 1D-CNN to detect DGA and enables binary and multiclass analysis of DGA. Berman [25] uses 1D-CNN with a convolutional layer of one-dimensional data to detect DGA. Chen et al. [26] apply 1D-CNN and BiGRU to detect DGA.

Shahzad et al. [27] uses a recurrent neural network to detect DGA outputs on a per-domain basis using the domain name only, with no additional information, which the authors compare to the performance of a DGA classifier based on the following RNN architectures: Unidirectional LSTM network, bidirectional LSTM (Bi-LSTM), and Gated Recurrent Unit (GRU).

Zhang et al. [28] design and implement several DGA classifiers based on machine learning (SVM and RF) and deep learning (CNN, LSTM, and Bi-LSTM) methods. Yang et al. [29] exploits the character-level characteristics of the DGA domain names and proposes a heterogeneous deep neural network framework that includes 1D-CNN and LSTM. LSTM has been used to detect binary DGA and multiclass DGA by the alphanumeric domain name [30, 31]. Tran et al. [32] present a new LSTM algorithm to address the multiclass imbalance problem in DGA-related botnet detection. Strategies with unbalanced datasets are vital for multiclass DGA detection. Balakrishna et al. [33] use an LSTM, which is adapted to predict better if the dataset is unbalanced. Josan et al. [34] use a bidirectional LSTM network for binary DGA and multiclass DGA detection. Other applications that use LSTM to detect DGA are discussed in works [35–38].

Liu et al. [39] combine a convolutional neural network and a bidirectional long short-term memory network to detect DGA. Yun et al. [40] show a method based on natural language processing and Wasserstein Generative Adversarial Networks and a new way to prevent attackers’ evasion of neural network’s DGA detection.

Malware detection is relevant for IoT applications (Internet of Things), and DGA detection for this IoT scenario has been investigated [41].

Li et al. [42] make the inference using the Hidden Markov Model (HMM). Koh et al. [43] uses a pre-trained context-sensitive word embedding to classify DGA. Cucchiarelli et al. [44] use the Kullback-Leibner divergence and the Jaccard Index to estimate similarities to detect DGA. Finally, Yilmaz et al. [45] use a method to detect DGA using LSTM and add a GAN (generative adversarial network) to infer previously unknown malicious domains.

Yu et al. [9] comment that simpler architectures are faster in training and inference and are less prone to overfitting. This conclusion is fundamental and was the focus of this work, as we seek lighter architecture toward achieving greater performance in protocols with MPC. Another work from Yu et al. [8] proposes heuristics for automatically labeling domain names monitored in real traffic. This labeled data is essential for improving the accuracy of deep learning models in detecting DGA. Finally, Yu et al. [10] propose a new way to label a large volume of data collected from real traffic as DGA-related and non-DGA-related, allowing models to be trained with large amounts of real traffic.

Sivaguru et al. [46] strengthen DGA detectors against adversarial attacks and evaluate deep learning models and random forests (RFs) to detect DGA using information beyond the domain name.

### 2.2 Secure multi-party computation for DGA detection

The work of Drichel et al. [16] is the one that is mostly related to our proposal. That work proposes using MPC protocols to classify domains into DGA and non-DGA. They implement their proposals using several different MPC frameworks: PySyft, TF-Encrypted, MP2ML, and SecureQ8 applied in the classifiers Inline [10], NYU [9], ResNet [6], and FANCI. However, despite its pioneering aspect, the work of Drichel et al. [16] still has several deficiencies:

- It uses CNN2D for performing private inference of DNS domains. This adds unnecessary complexity to the classifiers since 1D-CNN is best suited to text classification problems.
- It does not use privacy-preserving embedded layers. In practice, this assumes that *Bob* has to leak the embedding layer to *Alice* for her to perform the embedding operation in the clear. Thus, this solution is not end-to-end private.
- It does not consider LSTM models over MPC. Our work develops the first LSTM implementation available in any existing MPC framework.
- It considers only binary classification. We show how to carry out binary and multiclass DGA predictions.

### 2.3 Secure multi-party computation for natural language processing

Hao et al. [47] present private inference on transformer BERT based models in a client-server setting. Clients have private inputs, and servers hold proprietary models. One contribution is a customized homomorphic encryption-based method for matrix multiplication.

Adams et al. [48] present the first application of MPC protocols for CNN-based text classification. Their method adapts a CNN2D from the Crypten framework into a 1D-CNN by utilizing two 2D convolutional layers to emulate the behavior of a 1D-CNN. In contrast, our work directly implements a one-dimensional, private embedding layer, resulting in a more efficient solution. Furthermore, [48] focuses on word-level classifications, while our research emphasizes secure character-level text classification. Lastly, Adams et al. [48] does not address private embeddings. Their approach assumes that *Alice* first converts her text into an embedded vector using a publicly available BERT model before secret-sharing it. This method is infeasible for a model where the embedding is private and part of *Bob’s* confidential information. Our work overcomes this limitation by providing protocols and implementation for secure embeddings.

The model SecureNLP [49] has two security protocols for LSTM and RNN in the honest-but-curious model. The difference between SecureNLP and our solution is that we use LSTM for inference with characters, and SecureNLP conducts inference on words. Additionally, SecureNLP does not work with private embedding layers.

Knott et al. [50] offers a comprehensive overview of the Crypten framework, demonstrating its application in text classification, speech recognition, and image classification. In their work, text classification is conducted through a sentiment analysis experiment using a linear layer operating on word embeddings. Our approach differs from theirs in focusing on character-level rather than word-level classification. Moreover, their work does not involve private embeddings or provide an LSTM protocol and implementation.

## 3 Preliminaries

### 3.1 Domain generation algorithms

**Fig 2:**
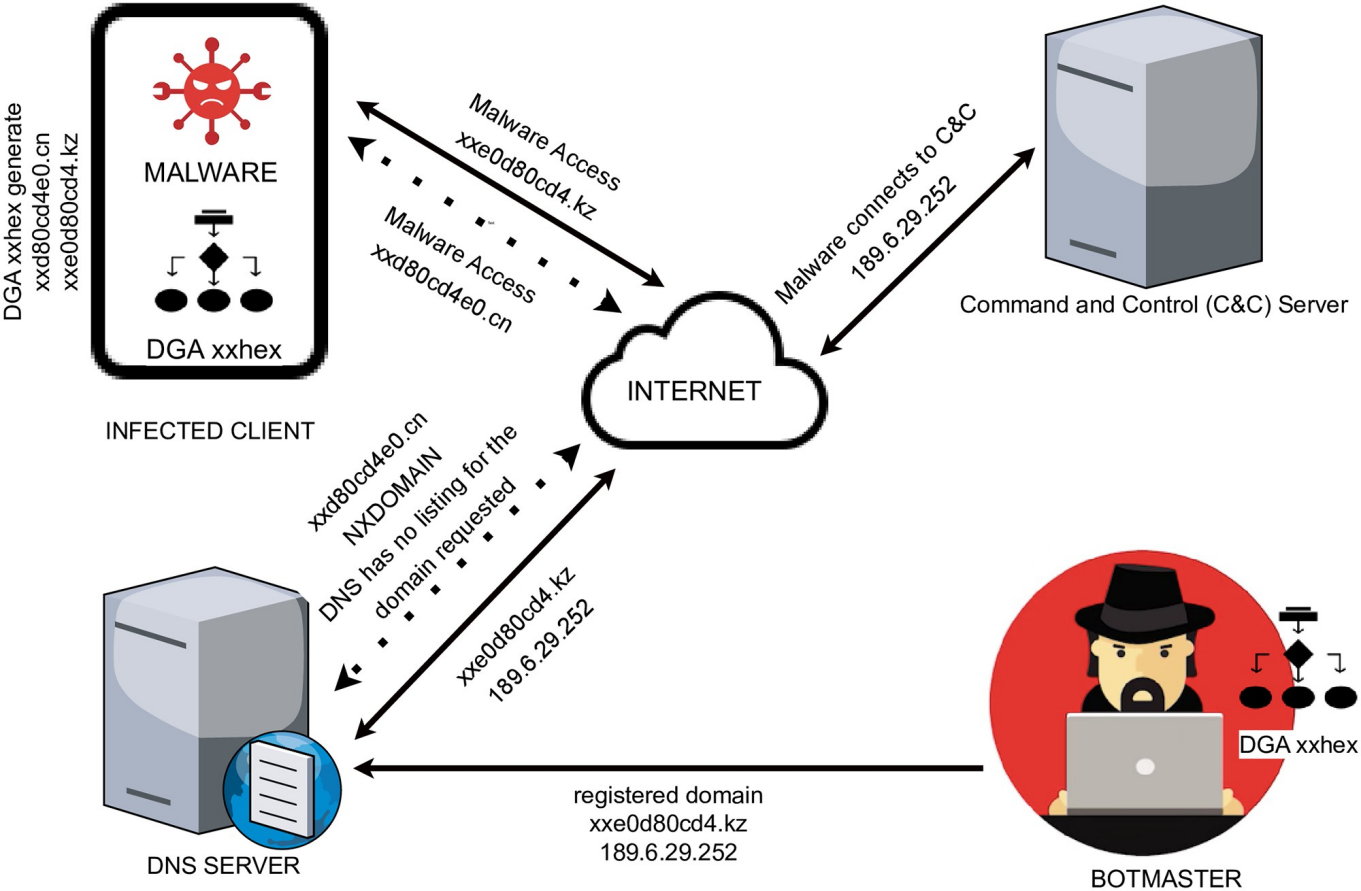
Illustration of the use of a DGA. The botmaster and malware on an infected client generate the same list of domain names. The botmaster registers a domain from the list. The malware attempts to resolve each domain from the list with the DNS until it finds the registered domain and a connection between the infected client and the C&C is successfully established.

A Domain Generation Algorithm is an algorithm that generates artificial malicious domain names. DGAs play a vital role in malware that relies on network communication between a botmaster and the bots (infected clients) [7, 9, 10]. As illustrated in Fig 2, the key idea is for a botmaster and malware on infected bots to independently run the same DGA with the same seed (e.g. based on the date) to generate the same list of artificial domains. The botmaster subsequently registers one or more of these automatically generated domains, while the malware on the bots attempts to resolve each domain with the DNS.

In Fig 2, for example, the botmaster and the malware on the bots all use the DGA “xxhex” to generate a list of domain names (pseudorandom strings of alphanumerical characters): “xxd80cd4e0.cn”, “xxe0d80cd4.kz”, etc. Out of these, the botmaster registers “xxe0d80cd4.kz” as the domain related to IP “189.6.29.252”. The malware on an infected client will then attempt to resolve each domain with the DNS. In the example in Fig 2, for domain “xxd80cd4e0.cn”, the DNS returns a Non-Existent Domain (NXDOMAIN or NXD) error, which indicates the DNS has no listing for “xxd80cd4e0.cn”. In contrast, for domain “xxe0d80cd4.kz”, the DNS returns IP “189.6.29.252”. Finally, the malware connects with IP “189.6.29.252”, where the command and control server is registered. Communication then proceeds between the botmaster and the infected machine.

The ability of the malware to dynamically generate and use new domain names prevents ordinary blacklisting from permanently blocking access between the botmaster and infected machines. Indeed, suppose a firewall or intrusion detection service detects and subsequently blocks one of these domains. In that case, the botmaster will use the DGA to create and register a new domain that is not yet blocked, and the malware will then use the same DGA as the botmaster to make a new connection. In this paper, we train and use neural networks that can distinguish such generated domains from real, benign domains. Such machine learning models are used to recognize DGA domains in DNS traffic and mitigate harm [8].

### 3.2 Neural networks

#### 3.2.1 Multilayer perceptron

A MLP is a machine learning model representing a fully connected neural network architecture. This model has an input layer, an output layer, and one or more hidden layers. Moreover, the neurons of adjacent layers are connected to each other.

Eq 1 describes an MLP neuron where the output, *y*, is derived from an activation function, *σ*. The argument for *σ* is formed by taking the dot product of the neuron’s input vector *x* with the weight vector *w* and then adding the bias scalar *b*.

$$\begin{array}{c} y\leftarrow \sigma (x\cdot w+b) \end{array}$$ (1)

The symbols *x* and *y* may be used in different contexts in subsequent sections of this paper. We will explicitly define their meanings each time they are referenced to prevent ambiguity.

#### 3.2.2 Long short-term memory networks

Understanding and abstract reasoning about a given piece of information depends on previous experience. For some tasks, such as reading text, humans can extract knowledge based on the context of previous and recent parts of the text. Standard neural networks do not have memory structures analogous to the human brain. This shortcoming is addressed in recurrent neural networks (RNNs), which closely resemble these memory processes. Therefore, with an RNN, it is possible to train and learn based on a previous element of an input sequence.

With RNNs, it is also possible to model problems where the input data is time-dependent or sequential, i.e., where the next task depends on the previous one. Examples of this can be found in activities such as temperature/weather forecasting, historical air quality trends, vehicle traffic congestion patterns, etc. Furthermore, in natural language processing, the meaning of texts and language structures humans produce depends on previous texts’ content.

Long Short-Term Memory Networks are a kind of RNN designed to resolve problems with vanishing and exploding gradients that occur with long dependencies in the traditional RNN models [51]. LSTMs are well suited for DGA detection [30–32, 34–37, 52, 53], hence we wish to study the effectiveness of LSTMs for the DGA detection problem when privacy is considered.

At their core, LSTM networks revolve around the concept of a cell state, a form of internal memory. LSTM cells have gates that regulate the flow of information into, within, and out of the cell. These gates can add or remove information from the cell state, acting as modification points in the memory system of the network.

We now describe such a cell in more detail and how it is used for inference, i.e., for computing outputs with a trained LSTM. We use the notation and closely follow the explanation presented in [54]. For our specific implementation, the input to the network is a sequence of characters *x*_1_, …, *x*_*t*_, …, *x*_*n*_. We refer to Section 3.2.4 for describing how each character is converted into a numeric representation. The inputs to the *t*-th cell consist of the output of the previous cell *h*_*t*−1_, the *t*-th character *x*_*t*_, and the state of the previous cell *c*_*t*−1_. The cell outputs *h*_*t*_, and its state is *c*_*t*_. We now describe how these quantities are computed. We now describe how these quantities are computed.

We first define how much of the previous state *c*_*t*−1_ we will “forget”, denoted by the parameter *f*_*t*_ in Eq 2. *f*_*t*_ is computed based on the dot product between the weights *w*_*f*_ and the concatenation of the output of the previous cell *h*_*t*−1_ and the cell’s input *x*_*t*_ and adding the result to the bias *b*_*f*_. Weights and biases are learned during training. This result is the input to the *sigmoid* function *σ* that returns values between 0 and 1. We will see that *f*_*t*_ equal to one means that we keep all of the previous state, while an *f*_*t*_ equal to zero means we forget everything about it when computing the state of the current cell.

$$\begin{array}{c} f_{t}\leftarrow \sigma \left(w_{f}\cdot \left[h_{t-1},x_{t}\right]+b_{f}\right) \end{array}$$ (2)

The state of the *t*-th cell is computed according to Eq 5. It is a weighted average of the previous cell state *c*_*t*−1_, and a state that depends on the current input *x*_*t*_, denoted $c_{t}^{\prime }$. The weights are *f*_*t*_ and *i*_*t*_, a coefficient that tells us how much of the “current” state ($c_{t}^{\prime }$) we want to keep. *i*_*t*_ and $c_{t}^{\prime }$ are computed according to Eqs 3 and 4. *w*_*i*_, *w*_*c*_ are weights and *b*_*i*_, *b*_*c*_ are biases computed during training. *tanh* is the hyperbolic tangent.

$$\begin{array}{c} i_{t}\leftarrow \sigma \left(w_{i}\cdot \left[h_{t-1},x_{t}\right]+b_{i}\right]) \end{array}$$ (3)

$$\begin{array}{c} c_{t}^{\prime }\leftarrow tanh\left(w_{c}\cdot \left[h_{t-1},x_{t}\right]+b_{c}\right]) \end{array}$$ (4)

$$\begin{array}{c} c_{t}\leftarrow f_{t}\cdot c_{t-1}\cdot i_{t}\cdot c_{t}^{\prime } \end{array}$$ (5)

Finally, the output of the *t*-th cell *h*_*t*_ is computed according to Eqs 6 and 7. *w*_*o*_ and *b*_*o*_ are weights and biases, respectively.

$$\begin{array}{c} o_{t}\leftarrow \sigma \left(w_{o}\cdot \left[h_{t-1},x_{t}\right]+b_{o}\right]) \end{array}$$ (6)

$$\begin{array}{c} h_{t}\leftarrow o_{t}\cdot tanh\left(c_{t}\right) \end{array}$$ (7)

*h*_*t*_, *c*_*t*_, and *x*_*t*+1_ are then fed into the next cell, and the computations happen similarly for the *t* + 1-th cell.

#### 3.2.3 Convolutional neural network

A Convolutional Neural Network usually comprises convolution blocks followed by a fully connected network. A standard convolution block consists of a convolution layer, followed by an activation layer, and then by a pooling layer. The standard convolution layer (2D-CNN) takes a 3D input of height *h*, width *w*, and depth *c* and consists of *f* number of 3D learnable kernels each of size *k* × *l* × *c*. Each kernel moves along two directions of the input to generate a 3D output. In a 1-dimensional CNN layer, the input is instead a matrix. The kernel moves along only one direction [55].

Let *x* be the input of a 1D-CNN. Let *y* be the output of the 1D-CNN layer, and *k* is the total number of kernels, where the length of *y* equals *l* − *k* + 1. The kernel applies a sliding window operation on the input *x*.

The output of a 1D-CNN can be represented by Eq 8, where *y*[*i*] represents the output in the position *i*. The operation involving *x* and *w* is a dot product; *b* is the bias; *w* is the weight of the 1D-CNN trained and represents kernels; *w*[*j*] is a kernel in the position *j*.

$$\begin{array}{c} y\left[i\right]\leftarrow \sum_{j=0}^{k-1}\left(x\left[i+j\right]\cdot w\left[j\right]\right)+b, \end{array}$$ (8)

#### 3.2.4 Embedding layer

Embedding layers make it possible to represent a text by using a vector of finite precision real numbers. In this work, each letter of a domain name is represented by a vector of finite precision real numbers. Instead of manually specifying the values for the embedding, they are trainable parameters.

We provide the first protocol and implementation for an embedding layer over MPC and implement it in the MP-SPDZ framework. The main idea behind our solution explained in detail in Section 5, is to represent Alice’s input to the protocol as a matrix *x* where each row of *x* is one hot encoding of a character of the domain name to be classified. So, each row of *x* consists of a binary vector with Hamming weight equal to 1. A dot product is made between *x* and the private embedding matrix *w*, resulting in *y*, and the result of the embedding layer is the input to 1D-CNN, LSTM, and MLP models.

Our process to get the output of the embedding layer can be described by Eq 9:

$$\begin{array}{c} y\leftarrow x\cdot w, \end{array}$$ (9)

Where the matrix *x* of dimension *l* × *c* represents one-hot-encoded inputs, the output of the embedding layer is the matrix *y* of dimension *l* × *d* representing an embedding of the input domain name to be classified, and matrix *w* of dimension *c* × *d* represents an embedding matrix, where *l* is the length of input domain, *c* is the length of the character set, and *d* is the size of the dimensional vector space.

Drichel et al. [16] proposed a solution for MPC-based private inference of DGA models. Their approach utilized several publicly available MPC frameworks for implementation. At the time, no protocol or implementation for private embedding existed, so the authors assumed that the embedding layer was publicly accessible and implemented in the clear by the party responsible for classifying the domain. However, this assumption could lead to information leakage about the model, especially if the embedding was trained on private data.

### 3.3 Privacy-enhancing technologies

#### 3.3.1 Differential privacy

Informally, a differentially private algorithm produces a given output with approximately the same probability, regardless of whether a single entry is present or absent in a dataset used to compute the algorithm output. This means that the output is negligibly affected by the participation of a single user, thereby offering privacy through plausible deniability.

We recall the definition of differential privacy:

**Definition 1** *(ϵ, δ)—Differential Privacy* [13]: *A randomized algorithm* M *with domain* D *is (ϵ, δ)—differentially private if for all S ⊆ Range(* M *) and for all* x,y∈D *such that* ||*x* − *y*||_1_ ≤ 1:

$$\begin{array}{c} Pr[M(x)\in S]\leq exp(\epsilon )Pr[M(y)\in S]+\delta \end{array}$$ (10)

Definition 1 implies that queries on datasets *x* and *y* differing on a single entry (||*x* − *y*||_1_ ≤ 1) should produce different results with probability bounded by a quantity depending on the parameters *ϵ* and *δ*. The constant *ϵ* is the *privacy budget*. The smaller the value of *ϵ*, the more privacy the randomized algorithm M offers. The constant *δ* captures a small probability of violating the privacy guarantee. When *δ* equals zero, we say we have pure DP. When *δ* > 0, we say we have approximate DP. *δ* is usually heuristically chosen to be smaller or equal to the reciprocal of the dataset size.

Deep learning models often leak information about their training dataset. Moreover, that leak is possible even when only black-box access is available, i.e., when an adversary only observes the output of the deep neural network. Differential privacy is used as a way to prevent such leaks. One can train deep learning models with DP guarantees using DP-SGD (differentially private stochastic gradient descent) [56].

The two essential steps in DP-SGD compared to traditional SGD are gradient clipping and noise addition. Gradient clipping is a technique that limits the magnitude of gradients to a predetermined threshold. Gradient clipping means the model’s gradients computed for each data point are scaled down if their magnitude exceeds a certain threshold. This step prevents individual data points from disproportionately impacting the model’s learning process, thus reducing the model’s sensitivity to any single data point. Once the gradients have been clipped, noise is added to provide privacy. The noise is typically sampled from a Gaussian distribution, with the standard deviation or scale parameter proportional to the chosen privacy budget *ϵ*. Smaller values of *ϵ* provide higher privacy but require more significant amounts of noise to be added to the model, which reduces the model’s accuracy.

To the best of our knowledge, we are the first to study the trade-off between privacy and the utility of DP-SGD in the context of DGA classification.

#### 3.3.2 Secure multi-party computation

Secure Multi-Party Computation protocols allow mutually distrustful parties to engage in a computation so that, at the end of the protocol, all the honest parties have received the correct output of the computation. No collusion of corrupted parties can learn any information other than what can be inferred from the inputs and outputs of the corrupted parties in the computation. We use a variant of the traditional MPC scenario, where computing servers offer MPC as a service, and the inputs can come from outside parties. These parties do not engage in the MPC protocol. We refer the reader to one of the many available introductions to MPC in the literature [14, 57–59].

The main idea behind all available MPC protocols is to decompose the function to be privately computed into a circuit consisting of addition and multiplication gates. Then, we execute the underlying MPC protocol for evaluating each addition and multiplication gate sequentially till the result is computed and revealed to a designated receiver. Decomposing the functions to be computed into circuits consisting of addition and multiplication gates is a non-trivial task. Efficient representations can dramatically increase the performance of an MPC computation of a given function.

We implement our solutions using MP-SPDZ [17], a publicly available framework for implementing multiple MPC protocols. MP-SPDZ has a high-level interface in Python for presenting a circuit to be computed over an MPC protocol. MP-SPDZ also has several circuit representations of machine learning algorithms, including multilayer perceptrons and 2D convolutional neural networks. However, no LSTM implementation in MP-SPDZ is available in the literature. Embedding layers are also not available in MP-SPDZ. This work presents novel circuit representations for computing the inference of LSTM networks and embedding layers.

We work with MPC protocols based on secret sharing. Secret sharing is the computational process of splitting a secret *s* into multiple secret shares *x*_*i*_ and giving these shares to shareholders. Only authorized families of the set of shareholders can recover the secret. Notably, no shareholder can obtain any information on the secret *s*. Secret sharing-based MPC protocols work by having the inputs to the computation secret shared among all the computing servers. Computations happen on shares rather than on the inputs themselves [14].

### 3.4 Quantization

The precision used to represent a machine learning model’s parameters impacts the model’s accuracy, runtime, and size. The performance impact of such precision is magnified when ML models are run on top of secure multi-party protocols. So, it is desirable to quantize (reduce the precision) used in ML models to make them lightweight. We aggressively use post-training float16 quantization to optimize our models [60]. By reducing the precision of our models’ weights to 16 bits, we reduce runtimes by approximately 23% to 42% in the 3PC setting (using replicated secret sharing), with a minimal impact on accuracy.

## 4 Proposed framework, privacy requirements, and threat model

### Overview of the proposed framework

We recall that *Alice* holds a DNS domain to be classified, and *Bob* holds a machine learning model that classifies DNS domains into malicious or benign. Our framework consists of set of *m* untrusted computing servers (MPC servers) $S=\{S_{1},S_{2},\ldots ,S_{m}\}$. While our proposed MPC protocols are general and work for any number of servers, we demonstrate our proposed protocols for *m* = 2 and *m* = 3. We assume pairwise authenticated and private communication channels between the servers. The communication between *Alice*, *Bob*, and any server $S_{i}\in S$, is also authenticated and private. Our proposed secure classification works as follows:

- Initially, *Alice* and *Bob* convert their real-valued private inputs (domains in the case of *Alice* and the model parameters in the case of *Bob*) into fixed-point representations. They then secret-share their respective fixed-point inputs with the computing servers.
- The computing servers then engage in MPC-based communications and computations to execute the MPC protocols to classify *Alice’s* domain names using *Bob’s* model.
- The inference result is secret shared among the computing servers at the end of the MPC protocols. The computing servers send their shares to *Alice*. Finally, *Alice* aggregates the secret shares and retrieves the classification result.

### Threat model

We build our MPC protocols on existing MPC primitives [17]. Our proposed protocols can be adapted to any threat model by replacing the underlying primitives available in the literature for the given threat model. We demonstrate how our protocols work for the case of “honest-but-curious” servers, which are participants that follow the protocol instructions but try to obtain as much information as possible about the secret data. We consider threat models: (1) with two and three computing servers; and (2) that withstand attacks by any adversary that can corrupt one server that he chooses, i.e., the privacy guarantees are maintained even when one of the MPC servers is corrupted.

### Privacy requirements

*Bob* cannot learn anything about *Alice’s* input. *Alice* should only learn the result of the classification protected by (*ϵ*, *δ*)-differential privacy and cannot learn anything else about *Bob’s* model and training data. The MPC servers cannot learn anything about *Alice’s* and *Bob’s* private inputs.

## 5 Methodology and proposed protocols

### 5.1 Training DGA classifiers using differential privacy

In our proposed solution, *Alice* is provided with the classification output of her domains. In the binary case, this is a label DGA or non-DGA. In the multiclass case, the output is either a label non-DGA or a label identifying one specific DGA family. This output should preserve the privacy of the individual entries of *Bob’s* training data set. We employ the well-known DP-SGD (differentially private stochastic gradient descent) technique [56] to mitigate privacy concerns and provide DP guarantees for *Bob’s* training data set according to Definition 1. We specifically train MLP, 1D-CNN, and LSTM models with DP guarantees.

Post-training quantization is an effective technique to improve the inference performance of a trained model. Post-training quantization transforms the model’s weights and activations from floating-point precision (32-bit) to lower-precision representations. We propose to quantize the trained model parameters to float16 [15], which causes minimal loss in accuracy and allows for wider deployment of machine learning models with various hardware specifications (e.g. GPU, CPU with float32 or float16 instruction sets). The DP guarantees follow for the post-training quantized model due to the post-processing property of DP. We note that our framework can be adapted to other quantization schemes as well [60–62]. By combining DP and quantization techniques, we can preserve privacy for *Bob’s* training data set and enable faster inference.

### 5.2 Secure inference of DGA domains

During the inference stage, *Alice* has an instance (raw text input, i.e., the domain name string) that needs to be classified as a DGA or non-DGA domain. *Alice* begins by adjusting the length of the given input text to a publicly known value *l* by truncating the input text or padding the input text with zeros. The fixed length text is then one hot encoded based on ASCII characters, resulting in a matrix *x* of dimension *l* × 128, where ASCII is a set of 128 characters. *Alice* then secret shares matrix *x* and *Bob* secret shares the model parameters with the computing servers. The architecture of *Bob’s* model is publicly known.

The computing servers execute MPC protocols that output the secret shares of the classification result to *Alice*. Our proposed framework is equipped to provide inference using models that use MLP, 1D-CNN, and LSTM architectures. All of these models have an embedding layer as the first layer. Next, we describe the proposed MPC protocols for these models that perform secure inference.

#### Basic building blocks

We build our proposed protocols upon a few building blocks already available in the MP-SPDZ framework [17]. We use MPC protocols $\pi_{\text{SIGMOID}}$ for the sigmoid function, $\pi_{\text{SOFTMAX}}$ for the softmax function, $\pi_{\text{MUL}}$ for secure dot product, $\pi_{\text{TANH}}$ for the hyperbolic tangent, $\pi_{\text{RELU}}$ for relu function, and $\pi_{\text{DENSE}}$ for dense layer. See [17] for a detailed description of these primitives.

#### Privacy-preserving embeddings

The embedding layer transforms the high-level input matrix *x* from *Alice* to provide a dense vector representation of the characters in the input text using *Bob’s* learned embedding weights *w*. Many previous works for MPC-based privacy-preserving text classification, including DGA detection, require *Alice* to embed the input text, which in turn requires the trained embeddings to be made public and may leak information regarding the training data unless trained with DP guarantees [16, 48]. Moreover, these trained embeddings may be proprietary. To mitigate the above scenarios, we propose a novel MPC protocol for the embedding layer to compute the embeddings of private input text in an oblivious manner. The vectors resulting from the embedding layer of our classification models represent the lexical information of the characters in the given DGA domain (URL) in the ASCII character set. The idea behind $\pi_{\text{EMBEDDING}}$ is simple: we extract the vector representation of each character in the input text (in our case, it is a domain or URL) from the trained embeddings with DP guarantees.

One of the simplest ways to extract such embeddings (Protocol 1) is to represent the input text as one hot encoded matrix *x* of dimension *l* × 128 and multiply it with the weights of trained embeddings *w* of dimension 128 × 128. The product is a matrix *y* of dimension *l* × 128 representing a set of vector representations of each character in the input text. We note that feature extraction done this way requires only multiplication operations for which state-of-the-art MPC primitives are available, which results in optimized performance of the MPC protocol for extracting embeddings of the input. Moreover, our protocol is general enough to work with character sets of arbitrary cardinality *c*.

**Protocol 1:** $\pi_{\text{EMBEDDING}}$ for secure inference of embedding layer

**Input:** Secret shared matrices *x* of dimension (*l* × *c*) representing one-hot-encoded inputs and *w* of dimension (*c* × *d*) representing embedding weights, where *l* is the length of the input text in characters, *c* is the cardinality of the character set, and *d* is the dimensionality of the embedding space.

**Output:** A secret shared embedding matrix *y* of dimension (*l* × *d*) of the input domain to be classified.

**1** $y\leftarrow \pi_{\text{MUL}}\left(x,w\right)$

**2 return** *y*

#### Privacy-preserving MLP

In our work, we leverage an existing implementation of an MPC protocol for secure inference with an MLP, available within the MP-SPDZ framework [17]. MLPs will be used as a baseline method in our framework.

The input is secret shared by *Alice*, while *Bob* secret-shares the model’s weights. Bob’s model in the architecture with MLP composes the weights of the embedding and dense layers.

#### Privacy-preserving 1-D convolution

We now present our solution based on 1D-CNN. Our architecture has an input layer with the protocol $\pi_{\text{EMBEDDING}}$, a layer with the protocol $\pi_{1\text{D}-\text{CNN}}$, and a layer with protocol $\pi_{\text{DENSE}}$ representing the dense layer in MPC. The input will be secret shared by *Alice*, while *Bob* secret-shares the model’s weights.

We leverage an existing proposal for a 1D-CNN [48] but provide our implementation [63]. The protocol $\pi_{1\text{D}-\text{CNN}}$ for secure inference with 1D-CNN is built upon (a) the existing MPC protocols available in the literature for $\pi_{\text{RELU}}$, and $\pi_{\text{MUL}}$ (b) our proposed MPC protocols for embedding $\pi_{\text{EMBEDDING}}$ and 1-D convolution $\pi_{1\text{D}-\text{CNN}}$.

Protocol 2 for secure inference with a 1-D convolutional layer takes as input (1) the secret shared embeddings obtained as output from Protocol $\pi_{\text{EMBEDDING}}$ and (2) the secret shared model parameters, i.e. weights of the kernels (*k* in total) each of size *k* for the 1-D convolution layer from *Bob*.

**Protocol 2:** $\pi_{1\text{D}-\text{CNN}}$ for secure inference with 1-D Convolution.

**Input:** The secret shared matrices *x* (obtained as the output of the private embedding computed by Protocol $\pi_{\text{EMBEDDING}}$, *b*, and *w* representing input, bias, and weight. The constants *k* and *l* represent the number of kernels and rows of *x*.

**Output:** A secret shared *y* of dimension *l* − *k* + 1.

**1 for** *i* ← 0 ***to*** *l* − *k* + 1 **do**

**2**  *y*[*i*] ← *b*

**3**  **for** *j* ← 0 ***to*** *k* − 1 **do**

**4**   $y\left[i\right]\leftarrow y\left[i\right]+\pi_{\text{MUL}}\left(x\left[i+j\right],w\left[j\right]\right)$

**5**  **end**

**6 end**

**7 return** $\pi_{\text{RELU}}\left(y\right)$

#### Privacy-preserving LSTM protocol

We now present our solution based on LSTM. Our architecture has an input layer with the protocol $\pi_{\text{EMBEDDING}}$, a layer with the protocol $\pi_{\text{LSTM}}$, and a layer with protocol $\pi_{\text{DENSE}}$ representing the dense layer in MPC. The input will be secret shared by *Alice*, while *Bob* secret-shares the model’s weights.

#### Protocol 3 describes the LSTM layer

The input for the LSTM layer is the secret shared output of the embedding layer, while *Bob* secret shares the kernel weights (*w*_*f*_, *w*_*i*_, *w*_*o*_, *w*_*c*_) and the biases (*b*_*f*_, *b*_*i*_, *b*_*o*_, *b*_*c*_). We refer the reader to Section 3.2.2 for an explanation of each one of these terms. The operations involve MPC protocols $\pi_{\text{SIGMOID}}$ for sigmoid, $\pi_{\text{MUL}}$ for secure multiplications, and $\pi_{\text{TANH}}$ for the hyperbolic tangent.

**Protocol 3:** $\pi_{\text{LSTM}}$ for secure LSTM

**Input:** Secret shared vector *x* (obtained as output of the private embedding computed by Protocol $\pi_{\text{EMBEDDING}}$), and secret shared values of the weights (*w_f_*, *w_i_*, *w_o_*, *w_c_*), and biases (*b_f_*, *b_i_*, *b_o_*, *b_c_*). The input length is publicly known. Let [a, b] denote the concatenation of *a* and *b*.

**Output:** A secret shared vector *y* containing the output after the LSTM layer of the inference process.

**1** *h*_0_ ← 0

**2** *c*_0_ ← 0

**3 for** *t* ← 1 ***to** n* **do**

**4**  $f_{t}\leftarrow \pi_{\text{SIGMOID}}\left(\pi_{\text{MUL}}\left(w_{f},\left[h_{t-1},x\left[t\right]\right]\right)+b_{f}\right)$

**5**  $i_{t}\leftarrow \pi_{\text{SIGMOID}}\left(\pi_{\text{MUL}}\left(w_{i},\left[h_{t-1},x\left[t\right]\right]\right)+b_{i}\right)$

**6**  $c_{t}^{\prime }\leftarrow \pi_{\text{TANH}}\left(\pi_{\text{MUL}}\left(w_{c},\left[h_{t-1},x\left[t\right]\right]\right)+b_{c}\right))$

**7**  $c_{t}\leftarrow \pi_{\text{MUL}}\left(f_{t},\pi_{\text{MUL}}\left(c_{t-1},\pi_{\text{MUL}}\left(i_{t},c_{t}^{\prime }\right)\right)\right)$

**8**  $o_{t}\leftarrow \pi_{\text{SIGMOID}}\left(\pi_{\text{MUL}}\left(w_{o},\left[h_{t-1},x\left[t\right]\right]\right)+b_{o}\right)$

**9**  $h_{t}\leftarrow \pi_{\text{MUL}}\left(o_{t},\pi_{\text{TANH}}\left(c_{t}\right)\right)$

**10**  *y*[*t*] ← *h*_*t*_

**11 end**

**12 return** *y*

## 6 Results

### 6.1 Dataset

The DGA dataset was obtained from DGArchive [64]. The dataset from DGArchive was the set containing all collected DGA examples up to 2019. We remove DGA families with low representation from the dataset (less than 30k samples). We end up with 1,000,000 examples from the DGArchive dataset for use as positive matches from the following families: bamital, banjori, bedep, beebone, blackhole, bobax, conficker, corebot, cryptolocker, darkshell, dircrypt, dnsbenchmark, dnschanger, downloader, dyre, ekforward, emotet, feodo, fobber, gameover, gameover_p2p.csv, gozi, gspy, hesperbot, locky, madmax, matsnu, modpack, murofet, murofetweekly, necurs, nymaim, oderoor, padcrypt, proslikefan, pushdo, pushdotid, pykspa, pykspa2, pykspa2s, qadars, qakbot, ramdo, ramnit, ranbyus, randomloader, redyms, rovnix, shifu, simda, sisron, suppobox, sutra, symmi, szribi, tempedreve, tinba, torpig, tsifiri, urlzone, vawtrak, virut, volatilecedar, xxhex.

For negative DGA matches, we have acquired approximately 1,000,000 domains from the last known version of the dataset “Alexa top 1 million domains” [65], which are used for training the model to recognize legitimate domains.

All data is shuffled and split into 80% for training and 20% for testing for binary and multiclass model architectures.

We convert alphanumeric characters representing domain names to lowercase for use in the model. Then, we convert each character to its corresponding ASCII code, which lies between the values of 0 to 127. Finally, the maximum length of a domain ASCII string is set to 64 characters. For domains whose size is less than 64, we prepend zero padding length to 64.

In this section, we evaluate our experimental results. First, we compare the 1D-CNN, LSTM, and MLP models using secure MPC when applied to binary and multiclass DGA detection with and without differential privacy. We also experimented with quantizing the models after the DP training (in which case, after training with DP, we applied quantization to reduce the weights to 16 bits and thus achieve performance gains in the inference phase with MPC).

### 6.2 Model architectures and parameters

All trained models have an embedding Layer as the first layer, where the input is a vector of 64 numerical elements resulting from transforming each character into ASCII code. The result of embedding is a 128 by 128 dimension array.

The last layer in all binary models comprises one dense layer with one neuron, activation function sigmoid, loss function binary cross-entropy, and optimizer Adam.

The last layer in all multiclass models comprises one dense layer with 65 neurons representing all families, the activation function softmax, the loss function sparse categorical cross-entropy, and the optimizer Adam with a learning rate of 0.001, batch size of 64, and 30 epochs.

We now provide further details about the other layers for each one of the architectures [63] we used:

- **MLP:** The binary and multiclass MLP models have a flatten layer to transform the data resulting from the embedding layer into one dimension data followed by a dense layer with 100 neurons, ReLU activation function and dropout with rate 0.1. The MLP binary model has 835,785 parameters, while the MLP multiclass model has 842,249 parameters.
- **1D-CNN:** The binary and multiclass 1D-CNN models have: (1) a 1D-CNN with filters = 32, kernels = 2, ReLU activation function and dropout with rate 0.1; (2) followed by a flatten layer to transform the output of the previous layer into one-dimensional data; (3) a dense Layer with 100 neurons, ReLU activation function, dropout with rate 0.1. The 1D-CNN binary model has 226,409 parameters, while the 1D-CNN multiclass model has 232,671 parameters.
- **LSTM:** The binary and multiclass LSTM models have: (1) a LSTM with 32 units with ReLU activation function and dropout with rate 0.1; (2) followed by a flatten layer to transform the output of the previous layer into one-dimensional data; (3) a dense layer with 100 neurons, ReLU activation function and dropout with rate 0.1. The LSTM binary model has 48,713 parameters, while the LSTM multiclass model has 55,177.

The DP-SGD parameters used in our experiments are available in TensorFlow Privacy [66] as follows: delta is 6.188 × 10^−7^, the clipping norm is 1, and the number of microbatches is 1. Regarding the noise multipliers, for an epsilon of 0.1, it is 2.51; for an epsilon of 2, it is 0.61; and for an epsilon of 5, it is 0.46.

### 6.3 Experiments

#### Utility-privacy trade-off

Table 1 provides a comprehensive analysis of the trade-off between utility (as measured by the accuracy of the model) and privacy as represented by privacy budget, denoted as *ϵ*, of the differential privacy mechanism) for all models.

**Table 1:** Results on the DGA inference accuracy for different noise levels with and without quantization.

Model	Non-Quantized, *ϵ* =				Quantized, *ϵ* =			
	0.1	2	5	∞	0.1	2	5	∞
1D-CNN binary	90%	93%	93%	99%	90%	93%	93%	99%
1D-CNN multiclass	25%	47%	53%	88%	25%	47%	53%	88%
LSTM binary	88%	91%	92%	97%	88%	91%	92%	97%
LSTM multiclass	23%	46%	51%	88%	23%	46%	51%	88%
MLP binary	90%	93%	93%	96%	90%	93%	93%	96%
MLP multiclass	24%	46%	51%	87%	24%	46%	51%	87%

The privacy budget *ϵ* is a quantifiable measure of privacy. As a rule of thumb, an *ϵ* value less than one indicates high privacy, a value between one and two suggests moderate privacy, while an *ϵ* exceeding two is considered low privacy. *ϵ* equal to infinity represents a model without any differential privacy guarantee. As expected, the model’s accuracy decreases as the privacy budget reduces, thus entering a higher privacy regime. The decrease is more severe for the multiclass case. This observation captures the trade-off between utility and privacy when applying differential privacy. Table 1 also presents the accuracy levels of each model when quantization is applied. The quantization step did not change the accuracy, even though quantized models are significantly faster than their non-quantized counterparts.

#### Runtimes

We implemented the MPC-based inference both in the scenario of two computing servers (2PC) as well as three computing servers (3PC) connected over a Gigabit Ethernet local area network. In the inference experiments, we used three Azure instances with 32 Cores of Intel(R) Xeon(R) Platinum 8272CL CPU @ 2.60GHz and 64GB of RAM. We used the following underlying MPC protocols available on MP-SPDZ [17] in our experiments: semi2k for 2PC and replicated secret sharing for 3PC. The runtimes are available in Table 2 and include communication and computation delays. The MLP model had the best runtimes, but 1D-CNN had the better accuracy, especially for higher values of *ϵ* (see Table 1).

**Table 2:** Inference using MP-SPDZ protocol.

Model	Setting	Inference Time (sec)	Rounds	Data Sent (MB)
MLP Binary	3PC	0.0778787	2773	17.217
1D-CNN Binary	3PC	0.319441	8551	21.6062
LSTM Binary	3PC	10.4153	195131	1485.53
MLP Multiclass	3PC	0.133239	3615	24.8676
1D-CNN Multiclass	3PC	0.359278	9382	29.0157
LSTM Multiclass	3PC	10.5449	197123	1489.92
MLP Binary	2PC	14.2051	42923	3951.16
1D-CNN Binary	2PC	14.2954	54151	3983.37
LSTM Binary	2PC	103.472	441023	26752.6
MLP Multiclass	2PC	14.577	44599	4054.73
1D-CNN Multiclass	2PC	14.6503	55831	4089.34
LSTM Multiclass	2PC	104.077	443895	26820.1

We note that runtimes are unaffected if the model is protected by differential privacy. Note also that runtimes strongly depend on the corruption threshold: the three party protocols (i.e., honest majority) are faster than the two-party protocols, in which there is no honest majority.

We also performed the same experiments regarding MPC-based inference but using the models we quantized after the DP training. The runtimes are presented in Table 3.

**Table 3:** Inference using MP-SPDZ protocol with quantization after DP training.

Model	Setting	Inference Time (sec)	Rounds	Data Sent (MB)
MLP Binary	3PC	0.0593449	2469	12.3028
1D-CNN Binary	3PC	0.223521	7371	12.3982
LSTM Binary	3PC	5.9959	137044	545.121
MLP Multiclass	3PC	0.076885	3038	15.0529
1D-CNN Multiclass	3PC	0.260958	7940	15.1483
LSTM Multiclass	3PC	6.15767	138573	546.706
MLP Binary	2PC	13.813	42723	3845.97
1D-CNN Binary	2PC	14.1394	51795	3937.96
LSTM Binary	2PC	60.3208	310659	15352.2
MLP Multiclass	2PC	13.9138	44599	3983.37
1D-CNN Multiclass	2PC	14.4177	54151	4054.73
LSTM Multiclass	2PC	60.247	312951	15388.2

Note that in the 3PC setting (using replicated secret sharing), the quantization step reduced inference runtime by approximately 23% to 42%. The reduction in execution time for the 2PC configuration (using semi2k) was 2% for MLP Binary, 4% for MLP Multiclass, 1% for 1D-CNN, and 42% for LSTM.

#### Comparison to previous results

The closest result to ours is the work of Drichel et al. [16]. There, they also propose a framework and protocols for private inference and classification of DNS traffic. Their runtimes and accuracy cannot directly compare to ours because of the weaker privacy guarantees they achieve (as pointed out in the related works section). Drichel et al. [16] leak information about the model to Bob (the embeddings). Moreover, their solution does not offer output privacy since there is no differential privacy training of the model. Despite these weaker guarantees, their solution is not faster than ours. All of their models have inference time higher than 1s, and their experiments do not consider network delays. In comparison, our most accurate classifier (CNN 1D) runs under 0.4 s, including network delays. Regarding communication complexity, all the models presented in [16] exchange over 190MB in messages. Our fastest protocol exchanges 17MB (with quantization), and our most accurate one exchanges 21MB (with quantization).

### 6.4 Security and privacy

Input privacy: The underlying MPC protocols from MP-SPDZ [17] that we use in our solutions (replicated secret sharing in the case of 3 computing servers with an honest majority; and the semi2k protocol in the case of 2 computing servers without an honest majority) implement a secure arithmetic black-box MPC. They only perform operations over secret shares, and no information is leaked during the computation over the secret shares. Moreover, we use MPC sub-protocols $\pi_{\text{SIGMOID}}$ for the sigmoid function, $\pi_{\text{SOFTMAX}}$ for the softmax function, $\pi_{\text{MUL}}$ for secure dot product, $\pi_{\text{TANH}}$ for the hyperbolic tangent, $\pi_{\text{RELU}}$ for relu function, and $\pi_{\text{DENSE}}$ for dense layer from MP-SPDZ. All these sub-protocols do not leak any information and are universally composable (UC) secure. The novel protocols that we propose ($\pi_{\text{EMBEDDING}}$, $\pi_{1\text{D}-\text{CNN}}$ and $\pi_{\text{LSTM}}$) therefore do not leak any information to the computing servers responsible for the MPC operations over the secret shares about *Alice’s* or *Bob’s* inputs; nor any private information is leaked to Alice or Bob other the result of the classification (which Alice can reconstruct by getting all shares of the output from the computing server). Our protocols UC-securely implement the ideal functionality $F_{PPCDGA}$ for privacy-preserving classification of domains as DGA or non-DGA that is described below for the case of classification using MLP, 1D-CNN or LSTM models.

Functionality $F_{PPCDGA}$

$F_{PPCDGA}$ waits until it receives as input from Alice her domain name *x* and as input from Bob his model *m* for DGA classification that he trained with DP-guarantees.

Upon receiving both inputs, $F_{PPCDGA}$ locally computes the result *y* of classifying *x* using model *m* and sends *y* as a public delayed output to Alice.

Output privacy: The guarantee that *Alice* does not learn information about individual entries used in the training of *Bob’s* model—is provided by the DP guarantees of DP-SGD [56] as utilized by *Bob*.

In a nutshell, DP-SGD guarantees (*ϵ*, *δ*) − differential privacy by repeated applications of the Gaussian mechanism. When training the model, Bob executes the following steps:

- Bob initializes the model *M* with random parameters.
- For each input *x*_*i*_ in a batch *B*, Bob computes the output of the model *M*(*x*_*i*_) and, using the corresponding label *y*_*i*_ associated with *x*_*i*_, computes the gradient of the loss function, denoted by *g*_*i*_.
- for each gradient *g*_*i*_ in the batch *B*, Bob clip the gradient if its L2 norm is larger or equal to a threshold *c*.
- Bob averages all the clipped gradients in Batch *B* and adds (to each coordinate of the gradient) a random variable sampled from a Gaussian distribution with mean 0 and a standard deviation *σ*.*c*, where *c* is the clipping threshold, and *sigma* is an appropriately chosen constant that ensures differential privacy.
- Bob then updates the model parameters using these noisy aggregated gradients times the learning rate.
- This process is repeated for each batch in the dataset.

The post-processing property of differential privacy ensures that the outputs of the model are also differentially private.

## 7 Conclusions and future work

This work presents the first framework for performing outsourced privacy-preserving DGA detection with input and output privacy. Input privacy means that no information about the domain being classified leaks to the computing servers or the model owner *Bob*, and no information about the model leaks to the domain owner (*Alice*) or the computing servers. Output privacy means that the output of the computation does not allow the extraction of individual entries in the training dataset of the ML model used in the framework.

We additionally proposed MPC protocols for LSTM and embedding layer in the MP-SPDZ framework, furthering the practical application of private and secure machine learning.

We compared the performance of CNN, LSTM, and MLP trained with DP and secure inference with various MPC protocols. The 1D-CNN presented better cost-benefit accuracy and performance on MPC for two parties, and in the case of the three computing servers, there were trade-offs between CNN and MLP.

We demonstrated that post-training float16 quantization with DP improved our runtimes by approximately 23% to 42% without a significant drop in accuracy when we were in the setting of three parties with an honest majority.

In a future work, we propose to apply the techniques of this paper to general tasks of natural language classification.

### Limitations and societal impacts

Our work only considers the actual performance against honest-but-curious adversaries. Considering fully malicious adversaries would increase our inference times, particularly for a dishonest majority setting. Developing protocols that perform well even for the malicious behavior case is left as an open problem.

In our adversarial model, we assume players input correct information into the protocol. Nothing in our solution prevents the players from presenting adversarial inputs as well as out of distribution inputs, in the case of Bob.

In terms of societal impacts, our solutions imply in a bit higher energy consumption, since, in order to achieve privacy, we have multiple servers performing the inference, rather than a single central server.

We have also not checked if differential privacy affects underrepresented types of domains in our data sets in a disproportional way. We leave that as an open problem, too.
